# Framing Concerns about Body Image during Pre- and Post-Surgical Consultations for Head and Neck Cancer: A Qualitative Study of Patient–Physician Interactions

**DOI:** 10.3390/curroncol29050272

**Published:** 2022-05-05

**Authors:** Maria Cherba, Boris H. J. M. Brummans, Michael P. Hier, Lauriane Giguère, Gabrielle Chartier, Hannah Jacobs, Véronique-Isabelle Forest, Alex Mlynarek, Khalil Sultanem, Melissa Henry

**Affiliations:** 1 Department of Communication, University of Ottawa, Ottawa, ON K1N 6N5, Canada; 2 Department of Communication, Université de Montréal, Montreal, QC H3T 1J4, Canada; boris.brummans@umontreal.ca; 3 Department of Otolaryngology—Head and Neck Surgery, McGill University, Montreal, QC H3A 0G4, Canada; michael.hier.med@ssss.gouv.qc.ca (M.P.H.); veronique-isabe.forest@mcgill.ca (V.-I.F.); alex.mlynarek.med@ssss.gouv.qc.ca (A.M.); 4 Department of Otolaryngology—Head and Neck Surgery, Jewish General Hospital, Montreal, QC H3T 1E2, Canada; 5 School of Psychology, University of Ottawa, Ottawa, ON K1N 6N5, Canada; lgigu023@uottawa.ca; 6 Department of Nursing, Oncology Division, Jewish General Hospital, Montreal, QC H3T 1E2, Canada; gchartier@jgh.mcgill.ca; 7 Department of Audiology and Speech-Language Pathology, Jewish General Hospital, Montreal, QC H3T 1E2, Canada; hjacobs@jgh.mcgill.ca; 8 Division of Radiation Oncology, Jewish General Hospital, Montreal, QC H3T 1E2, Canada; khalil.sultanem.med@ssss.gouv.qc.ca; 9 Gerald Bronfman Department of Oncology, McGill University, Montreal, QC H3A 0G4, Canada; melissa.henry@mcgill.ca; 10 Lady Davis Institute for Medical Research, Jewish General Hospital, Montreal, QC H3T 1E2, Canada; 11 Segal Cancer Centre, Jewish General Hospital, Montreal, QC H3T 1E2, Canada

**Keywords:** head and neck cancer, physician–patient relations, communication, body image, qualitative research, interaction analysis, framing

## Abstract

Patients with head and neck cancer report high unmet psychosocial needs as they undergo lifesaving treatments that can significantly alter their appearance and cause functional impairments. This qualitative analysis of recordings of 88 pre- and post-surgical consultations involving 20 patients respond to the need for empirical studies of patient–provider conversations about body image concerns. It indicates that the emphasis on concerns about survival, cure, and physical recovery during clinical consultations may leave concerns about the impacts of surgery on appearance and function unexplored and even silenced. The interviews with patients and medical team members that complement the analysis of the recordings suggest that an emphasis on survival, cure, and physical recovery can respond to the need for reassurance in the context of serious illness. However, it can also be problematic as it contributes to the silencing of patients’ concerns and to a potential lack of preparedness for the consequences of surgery. The results of this study can contribute to raising surgeons’ awareness of the interactional dynamics during clinical consultations. Moreover, the results highlight the unique role that surgeons can play in validating patients’ psychosocial concerns to support patients’ rehabilitation in both physical and psychosocial domains.

## 1. Introduction

Head and neck cancer is an umbrella term used to designate cancers starting in the head and neck region. They are often detected in advanced stages and are considered to have a poor prognosis [1]. In recent decades, there has been progress in reconstructive surgery and radiotherapy to reduce disfigurement and functional impairments following treatments. However, treatments can still have significant impacts on appearance and vital functions such as breathing, speaking, and eating [2,3].

Patients who undergo surgery for head and neck cancer are often concerned about changes in appearance and functional impairments. They report body image difficulties, higher levels of anxiety, and lower quality of life [2,4,5,6,7]. Changed appearance and functional impairments often become a barrier to social interactions and gaining back one’s identity [8,9,10]. These impacts may lead to severe distress and sometimes suicidal thoughts [9].

Patients may develop concerns about changes in appearance and functional impairments in the pre-surgical period when they anticipate the impacts of surgery and in the immediate post-surgical period as they experience acute changes [3,11]. As patients continue to recover, these concerns may resolve or persist, especially when surgery causes permanent changes [11,12]. While completing treatment and survival may be the primary focus of patients after being diagnosed with a life-threatening illness, concerns such as returning back to a normal life may become more prominent in the survivorship period [2,13,14,15,16,17,18,19]. Thus, during the pre-surgical period, patients are often concerned about the medical aspects of their care, and practical and emotional support services take on more importance during the post-surgical period as patients adapt to physical and functional changes [20,21].

Communication with health care professionals may impact patients’ experience of body changes resulting from head and neck cancer surgery, notably due to the differences between how patients and their treatment team view treatments and what aspects they find important to discuss [22,23]. These differences may lead to gaps in information provision and comprehension about potential surgical outcomes [24,25], such as patients’ unrealistic expectations about recovering function and returning back to normal [2,14]. Studies have also reported a lack of communication about body image, including disfigurement, as many patients do not disclose their concerns, and health care professionals may not know how to initiate and manage such conversations [15,20,26]. However, relatively little is known about how patients and care providers discuss changes in body appearance and function during real-time clinical interactions [4,15,27]. The goal of our study was to examine how surgeons and patients highlight or “frame” certain aspects of these changes during pre- and post-surgical consultations [27], as patients’ needs and preferences may change during their treatment trajectories [28,29].

## 2. Materials and Methods

The analysis presented in this article is part of a larger qualitative study of patient–medical team communication in surgical head and neck oncology, involving recordings of outpatient clinical consultations and interviews with patients and care providers. In this article, we focus on patient–surgeon communication.

### 2.1. Theoretical Framework

This study was guided by an interactional approach that examines care through conversations during medical consultations [30,31], as patients and care providers react and respond to each other’s actions [32,33,34,35]. According to this approach, concerns (such as changes in appearance and function) are co-constructed in interactions during medical visits: patient-initiated expressions of potential concerns offer opportunities for further elaboration and can be constructed as important or valid (for example, physicians can ask more questions or offer support in relation to some concerns, and patients can describe them in more detail), or not, in patient–provider conversations [36]. This approach can inform clinical practice by helping to understand “the organization of actual moments where patients make (or attempt to make) their concerns known to doctors” [37]. In addition, our analysis was informed by the concept of *framing*, which refers to the communication practices through which interaction participants, who may have different perspectives on the subjects they are discussing, foreground or emphasize certain aspects of their experience while backgrounding others [38,39]. In the context of patient–provider communication, this concept draws attention to different perspectives, goals, and needs that patients and care providers may have regarding the illness and its treatment [5,24,40]. This concept is helpful for identifying how these differences are expressed and addressed during consultations.

### 2.2. Participants and Recruitment

Adult (>18 years old) patients were eligible to participate if they (1) were diagnosed with a first occurrence, recurrence, or progression of head and neck cancer [41]; (2) were able to read and speak English or French; and (3) would be able to communicate through speech (or other audible modality compensating for voice loss) after surgery, as evaluated by the medical team. Moreover, patients were eligible to participate if the medical team identified surgery as a treatment option to be presented to the patient. Patients were not eligible if (1) their expected survival was less than 6 months, or they had poor functional capacity and prognosis [42,43]; (2) the medical team advised that the patient was not well enough physically or psychologically to participate; (3) patients were accompanied by a family member or another person who did not agree to participate.

Patients were recruited from a university-affiliated hospital in the province of Quebec, Canada. MC recruited patients with the help of the nurse navigator, who identified eligible patients and informed them about the study. Purposive sampling was used to ensure variability of sex, age, and cancer site and stage. To recruit care providers, MC presented the study protocol to the medical team during one of their weekly meetings. Professionals regularly involved in outpatient clinics (surgeons, a nurse, speech–language pathologists, and dietitians) agreed to participate. Students, residents, and other care providers who were not present during that meeting but who participated in patient consultations were approached on an individual basis to obtain consent. One care provider did not wish to participate, and their conversations with patients were neither transcribed nor used in the analyses.

### 2.3. Data Collection

MC conducted the observations and recordings of outpatient consultations for a period of 20 months, using a small wide-angle camera and an audio recorder. While audio/video recording is potentially intrusive, its use has been acceptable in various clinical contexts [44,45,46,47,48,49]. Participants were informed in the consent form that they could stop the recording at any time or choose the audio-recording only option if they were uncomfortable with the video. Recordings started during pre-surgical appointments (if possible) and continued up to six months after the surgery.

In addition, to complement the analysis and interpretation of clinical consultations and to identify possible ways of improving patient care, MC interviewed patients and team members who took part in this study. Interview protocols were created to explore the above-mentioned framing process, that is, to identify how patients and providers foregrounded and backgrounded concerns during pre- and post-surgical consultations. Patient interviews covered their care trajectory at the clinic; body changes that they had been experiencing; their perspectives on their interactions with the medical team; and suggestions for improvement. Interviews with the members of the medical team were structured around three themes: what they found important to discuss with their patients at different moments during the treatment trajectory; how they discussed particular body changes; and how they saw their role and the role of their colleagues in discussing these questions. The interview protocols can be found in Appendix A. Patients were interviewed before their surgery and approximately three and six months after the surgery (the dates were flexible to accommodate participants’ availability and physical state). These time points were chosen to allow patients to recover from the operation, so they could reflect on their experience over a longer period of time. Patients could choose the place most convenient to them. Most of the interviews took place in patients’ homes, and sometimes in a private meeting room at the hospital. Team members were interviewed in their offices at the hospital.

### 2.4. Data Analysis

To investigate how patients and surgeons framed changes in appearance and function, we used an interaction analysis approach, focusing on the sequential organization of conversations and the ways in which patients and care providers react to each other’s communicative actions/expressions [34,50]. The recordings of outpatient consultations were reviewed to identify sequences of communication around changes in appearance and function, following patient-initiated expressions of potential concerns, which were then transcribed verbatim and anonymized. All interviews were also transcribed verbatim. MC repeatedly examined and compared the consultation transcripts to explore emerging patterns of interaction (i.e., how patients and providers foregrounded and backgrounded concerns) and concurrently conducted a thematic analysis of the interview data [51]. Comparing the themes emerging from the analysis of consultations with those emerging from the analysis of the interviews helped focus the research on the aspects of patient–provider conversations around body changes that were significant to patients and providers (such as concerns, challenges, and suggestions for improvement). MC progressively discussed data analysis with BHJMB and MH. In addition, to validate emerging findings, a data analysis session around several selected excerpts from the transcripts was organized with professors and graduate students at the Department of Communication at the Université de Montréal.

## 3. Results

We audio- and/or video-recorded pre- and post-surgical consultations of 22 patients with head and neck cancer. Patients’ profiles are described in Table 1.

Eighty-eight consultations (including 19 pre-surgical) were recorded, lasting 37 min on average (from seven minutes to over two hours; median 31 min., mode 34 min.); longer meetings sometimes included medical procedures (e.g., changing a voice prosthesis, cleaning a wound, putting on a bandage) as well as longer wait times between consultations with different team members. Forty-five interviews with patients were conducted, lasting one hour on average. In addition, six interviews were replaced by a written questionnaire in situations where patients did not feel comfortable speaking or had not yet recovered their speech. Interviews with six team members (three surgeons, a nurse navigator, a speech–language pathologist, and a dietitian) lasted approximately one hour each.

Our analysis revealed two recurrent ways of discussing changes in appearance and function that patients and surgeons also described during the interviews as potentially challenging. First, we analyze how patients’ concerns about appearance and function were discussed in relation to survival and cure. Second, we analyze how patients’ descriptions of their experiences of changes in appearance and function were discussed in relation to surgical procedures and physical recovery. In addition, we examine patients’ and surgeons’ perspectives on the role of surgeons in exploring and responding to psychosocial concerns, and we examine how these concerns may manifest in patient–provider conversations. We present excerpts from consultation transcripts to illustrate these findings, along with excerpts from interviews. The data were collected in English and in French. French excerpts were translated into English. Transcripts were slightly modified to protect patients’ confidentiality when reporting qualitative data [52,53].

### 3.1. Emphasizing Survival and Cure in Response to Patients’ Expressions of Potential Concerns about Appearance and Function

Cancer is a life-threatening disease, and survival and cure are important concerns for patients and care providers. During the pre-operative visits, patients often asked about the date of the surgery. In the interviews, they reported concerns about survival and a sense of urgency to remove the cancer and prevent it from spreading. For example, they said:

*“[…] the question that I asked when I saw [my surgeon] for the first time was: ‘Will I live?’*”

*“[…] when I saw the surgeon, the goal was to have an appointment for the surgery as soon as possible. It was clear in my mind, that’s what was needed.”*

*“[…] one of the things I worry about is, all the time, between now and surgery day, is the cancer getting worse in there, or what does that mean, you know? I don’t know how fast this cancer moves in there.”*

Team members also talked about survival as being a primary concern for their patients, as well as for them as health care professionals. Some team members explained that communication to prepare patients for the consequences of surgery could be challenging in the pre-surgical period when patients are concerned about survival. As one team member said, for example:

*“[…] pre-operatively […], patients are, as much as they want to hear about […] how they are going to talk and swallow afterwards […] they have cancer, they want to get rid of it, and this is what their goal is. Their goal is to get rid of the cancer and once they get through that, then we can start focusing more on, not just getting rid of the cancer, but giving them their quality of life back.”*

The analysis of patient–provider conversations during the pre-operative visits indicates how the emphasis on survival may be reflected (and also constructed) in patient–provider conversations. We identified several moments in the transcripts where survival and cure tended to be foregrounded in response to the patients’ expressions of potential concerns about appearance and function (which, in turn, become backgrounded), as illustrated in Excerpt 1 below. This example shows how the patient brings up his concern about the consequences of the surgery and the choice of treatment by initiating the topic (01–03) and by pursuing it during the visit (13). It also shows how the surgeon emphasizes the uncertainty of cure without surgery (04) and how the patient and the surgeon arrive at a common understanding of their goal at the end of the conversation: to pursue the treatment option that provides the patient with a better chance of cure and survival (11–17). The surgeon acknowledges the patient’s concerns about the impacts of the surgery on his face (04), but the patient does not explicitly describe his concerns, and the surgeon does not explore this in more detail.

**Excerpt 1.**

[Following the first pre-surgical consultation when the surgery was explained to the patient and when he signed his consent for the surgery, the patient asked for another pre-surgical appointment with his surgeon. The goal of this appointment for the patient was to ask more questions about the impacts of the operation. He wanted to confirm his decision to choose surgery involving facial nerve sacrifice rather than radiotherapy as a treatment option. In the following excerpt, which takes place at the beginning of this second pre-surgical meeting, the patient mentions the potential impacts of the surgery on his face.]

*(01) PT: That’s because I also look at the disadvantages of the surgery.*

*(02) SUR: Good idea.*

*(03) PT: For the rest of my life, I will have an eye… I will be… it’s important on the level of the face, there will be changes… So… I try to balance… What would be best? It’s the surgery, but there are more serious consequences…*

*(04) SUR: Definitely. This is not an easy decision. And-and-and… Not to… not necessarily to help you take a decision, but we have a situation exactly like this with [another patient], almost the same situation. She chose radiation before, because the operation with the sacrifice of the facial nerve was too difficult for her to accept. And the radiation, it’s only been six-eight weeks since the radiation is finished, and now there is clear evidence of the tumor that remains […]*

[At the end of the meeting the patient summarizes the discussion by once again going back to his initial question about his treatment choice:]

*(11) PT: But thank you for what you have explained to me.*

*(12) SUR: My pleasure.*

*(13) PT: It confirms, when I came in, I really was not sure whether I would take the surgery or do radiation before, but from what you have told me, you have like, convinced me to take the surgery, to have a better life maybe as well… For the years I have left.*

*(14) SUR: Exactly. If your goal is the years, the time, you want to give yourself the best chance of…*

*(15) PT: Of life.*

*(16) SUR: To fight, and to successfully fight this, to win this battle.*

*(17) PT: Yes.*

Survival and cure remain important concerns for patients and medical team members after the surgery, especially in the first weeks when patients are recovering from major surgery, often involving reconstructive procedures. During this time, they are waiting for the results of pathology tests to know whether their tumor was completely removed and whether they need further treatment. In the interviews, some patients explained that they did not discuss changes in appearance and function during the post-operative visits because they were mainly concerned about the success of the surgery:

*“[…] So essentially, now, the communication [with my surgeon], is not, I’ll tell you [about my concerns] […] no-no, now it’s, OK, the scan, did you see something, you didn’t see anything, thank you, goodbye, I’m leaving, that’s all I want to know. The rest, I am managing […]”*

*“[…] the first thing is, above all, the lethal side of this thing, we agree, the question that is important for the doctor and for the patient, to know whether I still have my cancer, whether this cancer is expanding or regressing, whether it will be cured, what are my chances of cure, and well, this would be the first thing, it’s vital.”*

However, some patients did bring up concerns about appearance and function during post-surgical consultations. As in the pre-surgical period, we identified situations in which survival and cure were emphasized in response. Excerpt 2 provides an example of such an interaction, where survival and cure were framed as the main concerns, and concerns about appearance and function were backgrounded.

**Excerpt 2.**

[The patient comes to the clinic one month after her operation. As a result of the surgery, the patient lost her upper teeth and her face is still very swollen. The same morning, the patient had an appointment in the radiotherapy clinic, because she had to go through radiation and chemotherapy as additional treatment. At the beginning of the meeting, the patient meets with a resident physician who examines her and then calls in the staff surgeon. The surgeon examines the patient. They then discuss her upcoming radiotherapy, as well as other subjects, such as pain control and wound care. The excerpt starts when the patient shares her concerns about her appearance with the surgeon:]

*(01) PT: Because when I look at my face, I’m so depressed. ((Laughs a little.))*

*(02) SUR: I know. It’s going to change some more, you know.*

*(03) FAMILY MEMBER: She’s laughing, but*

*(04) SUR: I know, I know, it’s not, I know. No, you are not there at all, you are still swollen, so it’s just, which we wanted to, remember, I told you, I’m going to make you, I told you*

*(05) PT: OK.*

*(06) FAMILY MEMBER: She’s not happy about it now.*

*(07) SUR: I know, I know.*

*(08) PT: I just, I thought I would have a lot of scars, but, nothing like this*

*(09) SUR: Like this, yeah, the swelling.*

*(10) PT: Yeah.*

*(11) SUR: But this is going to, you’ll see, wait.*

*(12) PT: OK.*

*(13) SUR: You’re not there yet. Most important is to get rid of the cancer. And we’re still worried about that.*

*(14) PT: Yeah, I know.*

*(15) SUR: Remember, it was aggressive, it was going deep, and now you need that radiation.*

*(16) PT: But getting rid of the cancer looking like that?*

*(17) SUR: I know.*

*(18) PT: I can’t go out. Is that better?*

*(19) SUR: Yep. I know. Wait, we’re not, radiation, you’ll see, it’s another hit.*

*(20) PT: OK.*

*(21) SUR: We hit you once, and now the radiation is going to hit you again, it’s not*

*(22) PT: Oh.*

*(23) SUR: No, it’s true.*

*(24) PT: OK.*

*(25) SUR: It’s not easy, it’s not easy to go through all this. And you’re in the middle of it still.*

*(26) PT: OK.*

In this excerpt, the patient expresses a specific concern—that she is depressed when she looks at her face (01), which is further emphasized by the family member (03,06). The surgeon acknowledges the patient’s concern about appearance (“I know, I know,” 14–19) and offers reassurance as to the improvements that the patient will see over time (02,11). At the same time, the surgeon is also very direct in his description of what constitutes the most important concern at this time—“to get rid of the cancer” (13). The patient’s acknowledgments (“OK, OK”, 20–26) further contribute to framing cure as a priority.

In the post-operative interviews, some patients explained that the emphasis that was placed on survival and cure during the pre-surgical period was understandable in the context of a life-threatening illness, but it did not allow them to prepare for the consequences of surgery:

*“The social, psychological, functional aspects [should be addressed], but yes, I understand that, when you are in an emergency situation, you start by patching up the main water leak at home, then after you think about maybe redoing the entire plumbing system […] yes, there is an emergency situation and we are there to address it, you call the firefighters, you don’t have time to think about what color you will paint your living room walls in a year, six months, but yes, if you already have your answer […] it can help you get used to the idea of what is to come. So yes, it would be positive.”*

One patient specifically commented on how, in relation to concerns about survival, concerns about appearance may appear superficial:

*“And then you start questioning yourself, because everything that is related to aesthetics, I told myself, I refuse to be so superficial. But is it being so superficial? Or is it just normal that, hey, how will I be getting out of this, after this whole story? Will I have bandages for three months? Will I…”*

An excerpt from an interview with a medical team member reveals how this can become problematic in the post-operative period:

*“[…] I have patients that were telling me exactly that, ‘I was convinced that I did not care about appearance, but now I find that I look like a monster’. Because this also changes your perception of what’s important. When we, we have some sort of a balance, you tell yourself, I have a choice between living and dying, OK, well, and now, I live, but finally I look like a monster and I don’t like it, after all. I thought that I didn’t care about appearance, but no, I do care.”*

Overall, our analysis of the interactional data presented in this section shows how concerns about survival and cure that were reported by patients and medical team members during the interviews are also being foregrounded in patient–provider conversations during medical appointments. Consultation transcripts reveal moments in patient–surgeon dialogues when patients’ concerns about survival and cure (that tend to be backgrounded) can be explored.

### 3.2. Emphasizing Biomedical Explanations in Response to Patients’ Descriptions of Their Experiences with Changes in Appearance and Function

During the interviews, patients and surgeons explained that differences in how they experience changes in appearance and function were an important component of their communication during clinical consultations. This could pose challenges in situations where there may be “a disconnect” in their perspectives. As one surgeon stated,

*“[…] there is a patient that I saw, [he had] a transplant done [on the scalp], a good eight to ten months ago […] According to us, it was very nice, it had healed very nicely […] but he still did not have hair on his transplant. And then he asked, ‘Is it going to stay this way?’ And I, at the beginning, I didn’t understand, I told him ‘Well yes, it will pretty much stay like this […] but it’s very nice, it healed very nicely […]’ And he said, ‘Yes, but […] I don’t have hair there.’ And that’s when I understood what he meant […] when the patient asks us certain questions, we have a tendency to answer in a very medical way, whereas the patient […] his question ‘Is it going to stay like this’, we are not exactly answering it […]”*

In the post-operative interviews, some patients described how their awareness of such differences might lead them to avoid bringing up concerns they think are not relevant to their surgeons during consultations. For example, one patient explained why he did not share his concerns about going back to work as a teacher, due to changes in his ability to speak and articulate:

*“For me, it’s my personal life, it’s none of their business. […] No, I didn’t talk about it with [my surgeon]. According to me, it’s just, it doesn’t concern them, and then there’s no limitation […] The limitation, I’m the one who imposes it on myself, to say, I still have difficulties with speech, [my surgeon] can’t do anything and he’ll say, ‘But you, you can go ahead, come on, there’s no problem!’ For me it’s the image, I don’t feel comfortable to… face them, there’s always 15 to 20 people…”*

Our analysis of recordings of consultations revealed interactional moments when surgeons foregrounded biomedical explanations in response to patients’ descriptions of symptom experiences. Excerpt 3 below shows how a patient brought up his difficulties with eating and drinking over several post-surgical consultations. During the first post-operative visit, the difference between the patient’s and the surgeon’s perspectives first manifests when the patient brings up the sensation of his tongue (06–08) in response to the surgeon’s assessment that it is healing well (05). This difference then unfolds as the surgeon explains to the patient that even if he feels that his tongue is thick, it is not thick, and specifies why the patient feels that way (09,11,13). The patient’s potential concern is backgrounded or not explored in this conversation. In turn, the patient changes the subject and does not pursue the topic further (14). In his next post-operative visit, ten days later, the patient specifies why his tongue is bothering him—because it is more difficult for him to eat. Once again, the patient describes his feelings (15), and the surgeon explains the surgical procedures that caused the patient’s “impression” of having a swollen tongue (18,20,22), thus framing the conversation from a biomedical perspective.

**Excerpt 3.**

[Patient’s first post-operative visit, two weeks after his surgery for salivary gland cancer. At the beginning of his conversation with his surgeon, the patient tells him about how his tongue feels.]

*(01) SUR: Hello!*

*(02) PT: Hello doctor*

*(03) SUR: How are you doing?*

*(04) PT: It’s not too bad. ((SUR examines the patient.))*

*(05) SUR: It’s good, it’s healing well*

*(06) PT: The tongue, for me, feels very thick.*

*(07) SUR: Yes, because the sen-*

*(08) PT: And crooked.*

*(09) SUR: No, that’s because the sensation has changed, because the cancer was in the nerve and then we cut the nerve that causes the sensation, that’s why you feel like it’s big, but it’s not big.*

*(10) PT: OK.*

*(11) SUR: And the other thing is, it’s a nerve that causes movement in the tongue, that also, we had to cut it, that nerve, that’s why your tongue is a little crooked.*

*(12) PT: Yes.*

*(13) SUR: So these two things that you’re feeling, that it’s large and that it’s crooked, it’s because the sensations have changed. That’s why you’re feeling it this way.*

*(14) PT: Here, there is inflammation […] [changes the subject]*

[In his next post-operative visit, ten days later, the patient brings up the same concern about his tongue again, and further specifies why it is concerning to him—because it is more difficult for him to eat:]

*(15) PT: The sensitivity of my tongue, in the back here, I always feel that it’s thick, and I’m not eating on this side ((inaudible)).*

*(16) SUR: Stick your tongue out for me. ((Examines the tongue.)) So, the nerve that moves your tongue, when you stick your tongue out, it moves to the right.*

*(17) PT: Yes, I can feel it.*

*(18) SUR: OK, because the nerve that controls the movement on the right side of the tongue, it was affected by the tumor, it was wrapped, so it was necessary to take it out in order to take out the tumor. So, because of that, those are the changes for you for the tongue, elocution, eating, and it’s the reason why you feel like it’s really swollen.*

*(19) PT: Yes.*

*(20) SUR: Uh… We didn’t do any flap. ((Inaudible, briefly speaks to RES.)) So it’s only an impression, really, the tongue, it’s not really swollen, it’s not swollen at all.*

*(21) PT: Ah no.*

*(22) SUR: It’s an impression because of, and it’s, you’ll get used to it.*

*(23) PT: Ah yes, OK, because here it’s still really stiff and very ((touches his neck)).*

*(24) SUR: Ah that is, it’s the healing and scarring in the neck, but in the tongue, that’s the explanation.*

*(25) PT: OK. ((Continues to ask more questions about the neck.))*

One particular discussion topic that was identified in consultation transcripts and that illustrates the differences in patients’ and surgeons’ perspectives are the surgeons’ assessments of the surgical site as “beautiful” during post-operative visits that took place shortly after the surgery. Excerpt 4 shows an example of a consultation during which the patient met with a resident physician and two staff surgeons. When the patient shares that his wound does not look good to him (04), the resident offers an explanation (05), as in Excerpt 3. The patient acknowledges this difference in perspectives (“Of course I know the doctor is”, 06), as does the surgeon (“it looks bad but it is good”, 15), but the patient’s potential concern about appearance is not explored as the surgeons offer reassurance that it will improve (23).

**Excerpt 4.**

[This excerpt includes conversations from two post-operative meetings of a patient who had a tumor resected on one side of his neck. A skin flap was detached from his shoulder and attached to his neck to repair missing tissues. One side of the flap remained attached to the shoulder, so that it stayed connected to the blood vessels. After one month, when the resection on the neck had healed, the surgical team removed the flap and reattached it to the shoulder. The patient spent one month with the flap, which was covered with a big bandage. At the beginning of his first post-operative appointment, the patient meets with a resident who says, while she is examining the patient:]

*(01) RES: Your wound looks healthy and the flap is taking so nicely, it’s so beautiful.*

*(02) PT: Yeah?*

*(03) RES: Yeah, it looks good! ((Continues to examine the wound.)) Let me see here what we can do…*

*(04) PT: I, I glanced at it in a mirror and it didn’t look so good to me.*

*(05) RES: Well, because, you’re still*

*(06) PT: Of course I know the doctor is*

*(07) RES: No, it’s taken, the flap really well. Show me your teeth? Good. Close your eye. You don’t have eye symptoms or anything, no.*

*(08) PT: No.*

*(09) RES: OK, perfect, OK. I’m just going to get some ((Goes to the counter to get a pair of scissors. Goes back to the patient and continues to examine the wound.)) It’s so beautiful, it’s perfect! So you said, six more days of antibiotics?*

*(10) PT: Yeah.*

*(11) RES: Perfect. Let me speak to SUR and I’ll be right back.*

*(12) PT: OK.*

[When the resident comes back with a staff surgeon, the staff surgeon describes the wound in the following way after examining it:]

*(13) SUR: […] Well, it looks good but it’s… I mean, it looks bad but it is good.*

*(14) PT: Yeah, that’s what I said.*

*(15) SUR: It looks bad, but it is good. ((To RES)) What did you guys put in there? Nothing? ((Continues to examine the wound with RES.))*

[In this patient’s next post-operative meeting, a similar conversation took place:]

*(16) SUR1: ((Examines the wound.)) Beautiful, wow!*

*(17) PT: Yeah, I know, everybody says that*

*(18) SUR1: Well, it’s something relatively new, it’s not, the concept is not new, but… The actual flap itself is pretty unusual.*

*(19) PT: Yeah.*

*(20) SUR1: That’s good, it works beautifully for you, great. ((SUR2 enters the room.))*

*(21) SUR2: Hey! ((Looks at the wound.)) That looks great!*

*(22) PT: There you go, you’re going to tell me, it’s beautiful too. To me, it’s not beautiful! ((Laughs, everyone else laughs, too.))*

*(23) SUR2: It will be! Wait, we’ll see what it’s going to look like in a couple of weeks!*

In the post-operative interviews, patients reflected on such interaction patterns and talked about how such responses affected their experience. While noticing that some of their concerns were not fully acknowledged by their surgeons, some patients explained that they understood that physicians had a different perspective:

*“[…] when he said it was beautiful, I said, it didn’t look beautiful to me, you know, it’s not that I, you know, beautiful, that’s not the word to use; they could say, it’s coming along well, it’s going to be OK, it’s progressing, but beautiful ((laughs)), but that’s OK. […] I just got a joke out of it, that’s all. […] They are doctors and they are doing the best they can […] They do what they have to do and that’s it, you know, I don’t expect going there and getting moral support.”*

Some patients, however, expressed the need for more recognition of their concerns, experiences, and challenges by their surgeons:

*“When we meet with them [before the surgery], we understand that they don’t have the answers to everything. […] But on the other hand, after the operation, they could take a little time ((Inaudible, transcription from the notes: “take the time to sit down with the patient and explain everything, that would make a difference.”)) The trust that it would give. But now you feel like you’re a chunk of meat that was put on a cutting board and that needs to be fixed. It’s really the impression I have, a chunk of meat. So that, it doesn’t change anything in a physician’s life, but the impression that it gives to the patient, it’s ((inaudible.)) […]”*

*“[…] I’m not doubting the empathy there, I know that my physician, particularly, he was very-very empathetic, it’s someone who had, who was always in a very good mood, and I appreciated it very much […] But for them, there is nothing unusual, there is nothing unusual, patients like you, they see them all day long. So, when you go see them, you say, ‘Well, look, I have trouble eating, I have this,’—‘Ah yeah, that’s normal.’ Yeah, it’s normal, but do you understand that you eat three times a day and I have trouble eating two meals, do you understand? […] There’s nothing mundane about that, there’s nothing mundane about having your [body part] amputated, there’s nothing mundane about having trouble eating solid foods, there’s nothing… it’s not… it’s not mundane.”*

In their interviews, surgeons brought up similar suggestions to improve communication in situations where patients and surgeons may not have the same perspectives on body changes following surgery:

*“[…] I think that in post-operative follow-up we could improve this aspect […] Whether by listening a little bit more or sometimes by asking one or two more questions, to more precisely answer the patient’s question.”*

*“[…] you have to acknowledge the patient’s perception of, and I don’t say perception meaning it’s just a perception, I mean their observations, we have to acknowledge what their concerns are and address it and if it’s something that’s rectifiable or something that we can change, then we use every tool, but if there’s nothing we can do we just have to acknowledge it and recognize it and emphasize and support them.”*

In light of these comments, several opportunities for acknowledging the patient’s concerns and expressing empathy can be identified in Excerpts 4 and 5, right after the utterances in which the patient shares how his tongue feels and his difficulties with eating (see lines 07 and 15 in Excerpt 3) and when the patient mentions he did not like how his scar looks (see lines 04 and 22 in Excerpt 4).

### 3.3. The Role of Surgeons in Exploring and Responding to Psychosocial Concerns

In the interviews, patients and surgeons reflected on the role of surgeons in exploring and responding to psychosocial concerns, especially provided the limited amount of time surgeons have to meet with patients and the complex nature of surgical treatments. Patients talked about not bringing up concerns about appearance or the impacts of surgery on their social interactions. Surgeons explained that often they do not fully understand their patients’ psychosocial needs and rely on other members of the interdisciplinary team, such as the nurse navigator and the psychologist, to support patients in this regard:

*“[…] we have other resources [like the nurse navigator], but it’s not with [the surgeons], they are not there for an exchange about our emotions, and it’s okay, like I said. For them, it’s medical and… They barely get by, they’re always overwhelmed, like all the others.”*

*(Patient)*

*“They are super-technicians that solve a very precise problem. […] For me, what matters most with the surgery team it’s to know that they were doing the best possible job, that they were the best […] For that, they did their job, they met my expectations.”*

*(Patient)*

*“[…] we only have X amount of time, right, we’re dealing with complex situations, complex health care problems, complex cancer, complex psychosocial issues […] So I won’t deceive myself […] and say, ‘Oh we understand everything that this patient needs, clear cut, and we’ve addressed them all’. The truth is that we probably don’t understand their needs and we have not addressed them all. But I think that’s the reality of trying to practice health care in the setting that we work in and I think that we also rely a lot, we’re lucky that we have [our] nurse navigator […] social workers and our psychologist who help assess and recognize and address some of their needs beyond the direct immediate cancer care.”*

*(Surgeon)*

Especially regarding concerns about appearance, some patients explained in the interviews that they were not expecting to discuss them with their surgeons, as they understood that the surgeon’s role was related to other aspects of their care. Excerpt 5 below reveals how this role of the surgeon may manifest during patient–provider conversations in the post-surgical period.

**Excerpt 5.**

[This patient had a partial glossectomy: part of her tongue was removed and replaced with tissues taken from her forearm. During her first post-operative visit, she asked the surgeon about possible changes in the way her tongue looks as she continues to recover from the operation.]

*(01) PT: So after it’s going to be, how it’s going to be like, after the swelling is gone?*

*(02) SUR: After the swelling is gone, your speech will be almost normal if not normal.*

*(03) PT: But I’ll still see, like a*

*(04) SUR: No you’ll, again, you’ll see the color differential*

*(05) PT: Uh-huh.*

*(06) SUR: It’s never going to take on the color of mouth lining called mucosa, because it’s not mucosa, it’s skin.*

*(07) PT: Uh-huh.*

*(08) SUR: It’s always going to look more like skin then mucosa, because that’s what it is. But that will be just an appearance thing, because I expect that your articulation and your swallowing will be essentially normal.*

*(09) PT: Uh-huh. And also, I’ll lose the taste from this side, right. […] (12) PT: OK.*

[During another post-surgical appointment, the patient and the surgeon talk about the scars on the patient’s arm and neck that are taking longer to recover, and the surgeon is thinking about possible solutions to help with scar healing:]

*(10) SUR: […] the question is: How can we make your scars a bit better, you know?*

*(11) PT: OK.*

*(12) SUR: So I would try this first, we’ll try all the stuff that we know. At the end, if it doesn’t, if you still have red, we might send you to a dermatologist to see if they can*

*(13) PT: Yeah, because I think, my skin type is quite like, scarry*

*(14) SUR: You do, you scarred, yeah, because by now it shouldn’t be this red.*

*(15) PT: You see, like, all these scars*

*(16) SUR: Yeah, you just scar*

*(17) PT: It takes a long time to go, I know that, like, even just a mosquito bite, it takes a long time.*

*(18) SUR: Yeah, eh, yeah. So the good news there’s no cancer, the bad-, well*

*(19) PT: OK ((Laughs))*

*(20) SUR: The question is, how can we make you better with the scarring now, that’s the only thing*

*(21) PT: OK*

*(22) SUR: Which is a good thing, if we’re talking about scarring, that’s a good thing!*

*(23) PT: Yeah, yeah-yeah.*

When the patient asks a question about what her tongue will look like as she continues to recover from partial glossectomy (01,03), the surgeon describes visible changes as “just an appearance thing” (08) and emphasizes that the patient’s speech and swallowing will be “essentially normal” (02,08). In another post-operative meeting, the surgeon and patient discuss the scars on the patient’s arm and neck that are taking longer to recover. The surgeon acknowledges that the issue with scarring is “bad” (18), yet presents scarring as secondary to making sure there is no cancer (18, 22)—a statement with which the patient agrees (23), which further contributes to framing cure as the main concern. One patient reflected on such interaction patterns observed during post-surgical consultations in the following way:

*“[…] for me, I think it is both, [appearance] is as important as function, too, especially for a girl, for a female, I think, I’m definitely concerned about appearance, and so, yeah, I worry about it a lot, and I also asked questions about appearance, how it can go after, and that’s definitely something I’m concerned a lot about, yeah. For their response, it doesn’t make me feel anything, good or bad, uh… Yeah, because I know, it’s something that I have to check out by myself, they cannot really give you any surgeon answer about that.”*

Like other patients who were interviewed for this study, this patient shared her concerns about appearance during the interview, while also explaining that surgeons do not necessarily have a role to play in discussing with patients questions to which there are no “surgeon answers.” One of the surgeons interviewed for this study reflected on the possibility of surgeons playing a more proactive role in initiating and pursuing conversations about appearance:

*“[…] It doesn’t mean that we will be able to support the patient in this, but […] ask just one question […] ‘How are you, how are you dealing with […] the new appearance […]? Do you find it difficult […]? Sometimes my colleagues will tell me, we already spent a lot of time, but we ask if they have pain [and] […] we send them to the pain clinic when we are not able to help ease the pain. It’s the same thing in the end, it’s just another aspect. […] [We have] a role to play in this […] to say, ‘Listen, if you need it, it’s important, it’s just as important as the physical side, the control of tumor cells and all that.”*

Thus, the unique contribution of surgeons to patient care in this regard, as compared to the role of other team members such as the nurse navigator and the psychologist, is twofold: First, it could open the door for patients to express their concerns, as well as encourage patients to use available support services. Second, it could help frame concerns about appearance (as well as potential other psychosocial concerns) as legitimate and valid, to the same extent as concerns about curing cancer and physical recovery.

## 4. Discussion

Informed by interviews with patients and physicians, this study of in situ clinical interactions around changes in appearance and function contributes to oncology research in three ways. First, while previous studies have shown that many patients with head and neck cancer experience fear of mortality and may consider body changes as a trade-off for survival [2,13,18,54], our study empirically shows how survival and cure can be prioritized in relation to patients’ concerns about appearance and function during clinical consultations. In other words, our results underline the dynamic development of the patient’s understanding of body changes in patient–physician conversations. Treatment decision-making in oncology can be complex as patients and care providers balance quality of life versus length of life considerations [55]. While survival and cure are priorities for many patients, considerations such as the ability to maintain daily life tasks, breathe, eat, drink, and speak also shape decision-making and patients’ “sensemaking” [56] of treatment choices after treatments have been completed [57,58,59,60]. Patient priorities are also influenced by physicians’ input and recommendations for treatment [58,61]. Our study highlights this dynamic relationship between patient–physician conversations and the patients’ ways of approaching their illness by showing how, during pre- and post-surgical consultations, patients briefly bring up their concerns about changes in appearance and function, and surgeons do not explore them in more detail while emphasizing survival and cure. Our analysis of the interview data indicated that while many patients found this emphasis on survival reassuring (given the sense of urgency to remove the cancer and prevent it from spreading), some patients also felt that it did not allow them to prepare for the consequences of surgery. More particularly, they commented on how it led them to question whether their concerns about appearance were valid or superficial.

Second, our analyses revealed how differences in patients’ and surgeons’ perceptions of body changes were managed in patient–surgeon conversations and how biomedical explanations were emphasized in response to patients’ descriptions of their experiences. This can be problematic “because simply telling patients that their symptoms are not serious from a medical perspective does nothing to address the impact that these symptoms have on their lives” [62,63]. In addition, patients with head and neck cancer had described a perceived lack of empathy—especially situations in which “a physician seemed more focused on patient survival and what they considered to be surgical success than the patient’s struggle to cope with disfigurement,” as characteristic of a negative relationship with their care providers [2]. Interviews with patients and surgeons revealed that they were aware of the differences in their perspectives and that there was a need for more recognition of patients’ concerns, experiences, challenges, and the important impacts that the consequences of surgery had on their lives. Our analysis of clinical interactions identified examples of opportunities for acknowledging patients’ concerns during consultations.

Finally, our study provides insight into how patients and surgeons understand the surgeon’s role in exploring and responding to psychosocial concerns, as well as how this understanding can be expressed in (and also shaped by) patient–surgeon conversations during clinical consultations. While patients explained that surgeons do not necessarily have a role to play in discussing psychosocial concerns, our analysis of consultations provided examples of patient–provider interactions that may contribute to the patient’s understanding of the surgeon’s role. The lack of uptake of psychosocial concerns (compared with medical concerns) has been reported in many clinical contexts [64,65,66,67,68,69,70,71,72]. Through an in-depth analysis of how psychosocial concerns can be backgrounded while survival and physical recovery are foregrounded, our study highlights the unique role that surgeons can play in patient care: to help establish psychosocial concerns as legitimate and valid to the same extent as needs related to immediate cancer care. Future studies could examine whether a surgeon’s more active role in pursuing conversations about psychosocial concerns can help raise their patient’s awareness and reduce barriers to the use of support services. Previous studies have shown that many patients with head and neck cancer with clinical levels of psychological distress do not use available psychosocial services, or are unaware of them due to barriers such as patients not thinking that their distress is serious enough and stigma toward seeking care [73].

### 4.1. Limitations

The first limitation of this research pertains to the recruitment of patients for this study. Since the nurse navigator helped with patient recruitment and patients saw that MC knew the members of the medical team, there may have been a social desirability bias in that patients may have talked about their experiences in a more positive light. Moreover, since patients had to be physically well enough to participate in the study, our analyses did not reflect the experiences of patients who were very unwell, and for whom communication during clinical consultations may be more challenging. In addition, as most participants were born in Canada and had French or English as their mother tongue, the study does not reflect linguistic and cultural differences that may impact patient–provider communication. This research also does not include the perspectives of family caregivers who were present with the patients during appointments but were not interviewed as part of this project. Future research should consider their perspectives on communication about body image during clinical encounters. Finally, patients were cared for by an interdisciplinary team, and some had access to psychosocial support. Thus, the results of this study may not reflect the experiences of patients and care providers in settings with more limited resources.

### 4.2. Clinical Implications

Our results have several implications for clinical practice in head and neck oncology, as well as in other contexts where treatment can have important implications for patients’ appearance and function [3]. First, our results can heighten awareness among medical students and physicians of the interactional dynamics that contribute to emphasizing survival, cure, and surgical recovery while leaving unexplored psychosocial concerns related to changes in appearance and function. By being aware of how their responses to patients’ remarks about body image may foreground survival and cure, physicians can pay closer attention to turns of talk during consultations (how patients express potential concerns and how physicians respond), ask more questions to clarify what the patient’s main concerns are, and provide information about available support as needed. Put differently, by staying mindful of concerns that are important to patients as they emerge in clinical consultations, physicians “might best attend to what patients treat as meaningful for their lives and treatment” [74]. Helping patients explore “what matters most” to them [75] before and after surgery is important to achieve shared decision making and to provide care that is respectful of patients’ values and preferences [76,77,78] (for examples of strategies to explore patients’ body image concerns, see [79]).

Second, the results of the study can help physicians observe how the differences between their perspectives and their patients’ perspectives on changes in appearance and function can be expressed in clinical interactions. Thus, they can identify opportunities to acknowledge their patients’ experiences (for some specific strategies of empathic acknowledgment and examples of questions to discuss the differences between objective and subjective assessments, see [62,80,81]). The transcripts we analyzed for this study could be used to create case studies and simulation scenarios to help students and professionals practice such challenging conversations. Simulation-based communication curricula that have been developed in surgical head and neck oncology [82,83,84] could integrate specific scenarios focusing on discussions about changes in appearance and function.

Finally, the results of this study highlight the value of interdisciplinary care in head and neck oncology. They also highlight the unique role that surgeons can play in validating and normalizing patients’ body image concerns as well as in proactively initiating and facilitating conversations about such concerns in the context of life-threatening illness [79,81,85]. This conclusion is consistent with current literature that emphasizes the need for more integrated psychosocial care and the important role that surgeons can play as members of an interdisciplinary team in terms of screening patients for psychosocial distress, especially provided the high levels of unmet needs in this patient population [86,87,88,89]. Future studies could examine surgeons’ views of this role, explore ways to integrate this role into their clinical work, and identify what support they may need in this regard within the specific settings of their practice, as surgeons report the need for more support in relation to the psychosocial aspects of patient care [90].

## 5. Conclusions

This study reveals how psychosocial needs can be left unexplored and even silenced during conversations between surgeons and patients with head and neck cancer as survival, cure, and biomedical explanations are emphasized in response to patients’ expressions of concerns about appearance and function. The results suggest that surgeons could play a unique role in supporting the rehabilitation of patients in both physical and psychosocial domains. They can do this by espousing a more empathic stance that enables patients to be better prepared for surgery and readjust to the changes in their bodies through process-oriented, rather than avoidance-oriented or rationalization-oriented, communication. Our in-depth analysis of actual clinical interactions serves to stimulate discussions about potential changes in practice to make communication more patient-centered and focused on healing rather than on curing in accordance with the Hippocratic Oath.

## Figures and Tables

**Table 1:** Participant sociodemographic and medical profiles.

Sex	16 Men, 6 Women (n = 22 ^1^)
Age	Ranged from 30 to 89 years old, mean 65, median 65
Country of origin and language	18 participants were born in Canada, 2 immigrated to Canada; 14 participants spoke French and 8 spoke English.
Cancer site, stage, and treatment	Cancer sites included: larynx, jaw, hypopharynx, nose, tongue, salivary glands, floor of mouth, palate. A total of 10 patients were diagnosed with advanced stage cancer (stages 3 and 4), 7 with an early-stage cancer (stages 1 and 2), and 3 patients had an unknown primary tumor. For 14 patients it was their first head and neck cancer diagnosis, and for 6 patients a recurrence or a second cancer. A total of 13 patients had radiotherapy and/or chemotherapy treatments after their surgery.
Family members	During almost all consultations, close family members accompanied patients (partner, children, sibling, or parent). One patient came to appointments alone. A total of 11 patients were married, 3 were divorced, 2 were widowed and 4 were single.
Education and work status	13 participants had a high school or a college diploma, and 7 had a university degree. A total of 14 participants were retired, 3 were working part-time, and 3 were working or studying full time.
Family income	Ranged from 20,000–39,000$ to over 100,000 $/year, median 40,000–59,000 $/year.

^1^ One person stopped participation after pre-surgical consultation and one participant decided not to have surgery after pre-surgical consultation. For these participants, only demographic information about sex, age, spoken language, and cancer site was collected.

## Data Availability

The data are not publicly shared as per our ethical approvals, to protect the confidentiality of study participants.

## Appendix A—Interview Questions

**Interviews with patients**

**First interview (before the surgery)**

Thank you for taking the time to talk with me today. The goal of this interview is to explore your experiences during the consultations with your medical team that you have had so far. In particular, we are interested in your conversations with the team about the changes in your body resulting from cancer and surgery. We also want to know what was most or least helpful to you while coping with your diagnosis and preparing for the surgery. This will help us analyze the video recordings while taking into account your experience, so that the results of the study can be used to tailor the care to the needs of people followed at the Head and Neck Surgery clinic.

- To start, I would like to learn more about your care trajectory at the Head and Neck Surgery clinic so far.
  - How were you referred to the clinic? How were you diagnosed? How were you presented with surgery as a treatment option? And how did you decide to have surgery? How are you preparing for the surgery?
- Now, let’s talk more about the changes in your body you have been experiencing due to your diagnosis.
  - How would you describe the changes you have noticed in the appearance or the functioning of your body before you were diagnosed? Have they changed since? And what are you experiencing now?
  - What helps you cope with these changes? Has the medical team played a role in helping you to cope with these changes?
- I am also interested in knowing more about the interactions with the medical team you have had so far.
  - During the consultations that you have had, what were important things for you that you wanted to discuss with the team? Do you feel like you had an opportunity to bring up these subjects and ask the questions you had? Did you get the chance to express the concerns you had?
  - How would you describe your conversations with the team about changes in the appearance and functioning of your body related to your diagnosis? And the possible effects of the surgery on your body?
  - What information did you receive from the medical team about the possible effects of the illness and surgery on your body? If you have not received any information, what would you like to know?
  - And did you ever feel like you needed more information or support?
- Next, I would like to ask you about your experience of preparing for the surgery.
  - What are your expectations? What are your concerns? Have you talked about your concerns with the team? And how helpful/unhelpful were these conversations?
- To conclude, I have a few final questions.
  - Based on your experience, what suggestions would you have for the team to help people followed at the clinic in terms of adjusting to body changes due to the illness?
  - Based on your experience, what suggestions would you have for the team to help people followed at the clinic prepare for the surgery?
  - Would you like to add anything to what we have talked about today? Did we leave anything out that seems important to you? Is there anything that we have not discussed that you want to talk about?

Thank you for your participation in this study. We greatly appreciate your input.

**Second and third interviews (3 and 6 months after the surgery)**

Thank you for taking the time to talk with me today. The goal of this interview is to explore your experiences during the consultations with your medical team that you have had so far. In particular, we are interested in your conversations with the team about the changes in your body resulting from cancer and surgery. We also want to know what was most or least helpful to you while coping with your diagnosis and recovering from surgery. This will help us analyze the video recordings while taking into account your experience, so that the results of the study can be used to tailor the care to the needs of people followed at the Head and Neck Surgery clinic.

- To start, I would like to learn more about how your surgery went and how you have been recovering.
  - Would you please describe your experiences while staying in the hospital? What were the body changes you noticed? Has the medical team played a role in helping you to cope with these changes? What was helpful/not helpful in this regard?
  - And over the last [two] months, when you were discharged from the hospital and recuperating at home, what body changes have you been experiencing as a result of the surgery? How have you been adjusting to these changes? Has the medical team played a role in helping you to cope with these changes? What was helpful/not helpful in this regard?
  - Now that you have had the surgery, do you feel that there is anything that you would have found helpful to know before the surgery? Is there anything that surprised you or that you were not prepared to?
- Now, I would like to know more about the interactions with the medical team you have had since the surgery.
  - During the consultations that you have had, what were important things for you the that you wanted to discuss with the team? Do you feel like you had an opportunity to bring up these subjects and ask the questions you had? Did you get the chance to express the concerns you had?
  - How would you describe your conversations with the team about the process of recovery from surgery? What information did you receive from the team? Did you ever feel like you needed more information or support?
- Next, I would like to ask you some more questions about your recovery from the surgery.
  - What are your expectations? What are your concerns? Have you talked about your concerns with the team? And how helpful/unhelpful were these conversations?
- To conclude, I have a few final questions.
  - Based on your experience, what suggestions would you have for the team to help people followed at the clinic in terms of adjusting to body changes due to the illness and surgery?
  - Based on your experience, what suggestions would you have for the team to help people followed at the clinic recover from surgery?
  - Would you like to add anything to what we have talked about today? Did we leave anything out that seems important to you? Is there anything that we have not discussed that you want to talk about?

Thank you for your participation in this study. We greatly appreciate your input.

**Interviews with members of the medical team**

Thank you for taking the time to talk with me today. The goal of this interview is to explore your perspective on your communication with surgical head and neck oncology patients. Your responses will inform my ongoing analyses of patient interviews and observations of outpatient appointments at the clinic. I am particularly interested in your conversations with patients about appearance and functional changes following surgery, both during the pre-surgical and the post-surgical stages. I would like to understand better how you perceive this communication, what aspects you find challenging, and also what works well.

- To start, I would like to understand the trajectory that a patient goes through with you, before and after surgery. Starting from the first time when you meet with the patient, and then up to several years after the surgery, you have several conversations. In terms of communication, I am interested in what you find important to discuss with your patients at different time points in their trajectory.
  - During pre-surgical consultations, what is your approach to communicating about body changes? What are important things you want to discuss with patients? What subjects or areas do you usually cover? To what degree does this vary depending on certain patient characteristics?
  - According to you, what are the most important subjects or areas for the patient? To what extent do you think they are similar to the things you find important to discuss as a physician? How do you manage discrepancies, if any?
  - During post-surgical consultations, what are the most important things you want to discuss with patients? What subjects or areas do you usually cover? To what degree does this vary depending on certain patient characteristics?
- Now, let’s talk more specifically about your communication with patients about body changes, the process of recovery from surgery, and adjustment to body changes.
  - How do you address body changes following surgery and the process of recovery and adjustment in your pre-surgical consultations?
  - How do you address body changes following surgery and the process of recovery and adjustment in your post-surgical consultations?
  - How do you see your role, as well as other team members’ roles, in helping patients prepare and adjust to body changes following surgery?
  - How would you describe “good” communication? In your experience, what works well in terms of preparing patients to the surgery and supporting them as they recover?
  - And what are challenges when it comes to communicating with patients about the consequences of the surgery and the recovery process? How do you think these challenges might be addressed?
- Would you like to add anything to what we have talked about today? Did you leave anything out that seems important to you?

Thank you for your participation in this study. We greatly appreciate your input.
